# Ocean-colour anomalies quantified by the human eye

**DOI:** 10.1093/plankt/fbae027

**Published:** 2024-06-07

**Authors:** Robert J W Brewin, Giorgio Dall’Olmo

**Affiliations:** Department of Earth and Environmental Sciences, Centre for Geography and Environmental Science, University of Exeter, Penryn, Cornwall, TR10 9FE, UK; Department of Oceanography, Istituto Nazionale di Oceanografia e Geofisica Sperimentale - OGS, Borgo Grotta Gigante 42/c, 34010 Sgonico, Trieste, Italy

**Keywords:** ocean colour, phytoplankton, Secchi disk, Forel-Ule colour

## Abstract

Phytoplankton turn seawater green when their concentration increases. This allows us to monitor them using ocean colour. However, as the spectral properties of phytoplankton and their relationship with other coloured substances in seawater vary, subtle differences (anomalies) in ocean colour occur that can cause large errors in estimates of phytoplankton abundance. Identifying and understanding these anomalies is required to interpret ocean-colour data properly, but not all scientists have access to, or can afford, the *in-situ* instrumentation needed to do this. We show that practical, low-cost tools developed in the 19th century (a Secchi disk and Forel-Ule colour scale) can be used to quantify a colour anomaly in the Weddell Sea. Our findings imply that ocean-colour anomalies can be identified using affordable methods. Furthermore, records collected over the last century may contain clues on how ocean ecosystems have changed with climate.

## INTRODUCTION

Seventeenth-century navigators, like Henry Hudson, sailed through hostile icy waters with little more than a compass and their vision. They were acutely aware of changes in the colour and transparency of the ocean, noting that the water was bluer where there were icebergs, and they used this information to help guide safe passage (Wernand and Gieskes, 2011). We now know that the colour and transparency of the ocean are controlled by phytoplankton and other covarying coloured dissolved and particulate substances (Morel and Prieur, 1977). Phytoplankton modulate planetary biogeochemical cycles and have a profound effect on marine ecology by fueling the marine food web. They turn water green and reduce its transparency when their concentration increases. This has allowed modern scientists to study phytoplankton at synoptic scales using satellite ocean-colour measurements.

However, the blue signatures observed by early navigators in polar seas are not necessarily reflective of low phytoplankton concentrations because the relationship between ocean colour and phytoplankton concentration varies regionally, reflecting changes in environmental conditions and microbial ecology. In the early 1990’s, scientists noticed that, for a similar concentration of phytoplankton, the colour of the Southern Ocean appeared bluer than that of waters at lower latitudes (Mitchell and Holm-Hansen, 1991). This Southern-Ocean colour anomaly is thought to be related to the unique size and taxonomic structure of phytoplankton as well as differences in the contributions from coloured non-algal particles and dissolved substances (Robinson et al., 2021). Ocean-colour anomalies also exist in other regions. For example, the Baltic and Mediterranean Seas have higher concentrations of dissolved substances that absorb blue light preferentially over other colours, meaning the water appears greener than in other regions with similar concentrations of phytoplankton (Jerlov, 1955; Morel and Gentili, 2009). Identifying and quantifying these optical anomalies is key to the successful use of ocean colour for studying phytoplankton. But not all scientists have access to, or can afford, the *in-situ* instrumentation needed to do this.

Simple instruments developed in the 19th century and based on human vision could fill this gap. These include the Secchi disk that is used for measuring water transparency by lowering a white disk into the water and measuring the depth at which it disappears (Secchi, 1864), and the Forel-Ule colour scale—a scale of 21 shades of ocean colour (Forel, 1890; Ule, 1892)—used for measuring water colour. The Secchi disk and Forel-Ule colour scale are still used routinely today, for example, in participatory science projects and for low-cost environmental monitoring (e.g. George et al., 2021). They are engaging tools for teaching as the human participant is the sensor and, like former navigators, becomes connected with their environment in a way that is not possible using modern optical sensors.

## METHOD

To quantify whether ocean-colour anomalies can be identified by the human eye, we collected visual measurements of ocean colour on three cruises across the Atlantic Ocean, spanning low-latitude regions, and on a high-latitude cruise in the Weddell Sea, Antarctica. We used a 30 cm white disk attached to a profiling rig for measuring the Secchi depth and used the Forel-Ule colour scale of Novoa *et al.* (2014) for measuring the colour of water above the Secchi disk at half the Secchi depth. We collected data on all four cruises using consistent methods, as described in detail in Brewin *et al.* (2023). Briefly, at each station, the profiling rig was deployed (typically two casts), and the depths at which the Secchi disk disappeared and reappeared were measured. A wire-length sensor was used to determine these depths, although on some Atlantic cruises slightly different methods were used (with consistent results; see Section 2.3.2 of Brewin *et al.*, 2023). Forel-Ule colour scale measurements were collected by visually comparing the scale with the colour of the water above a background of the white disk at roughly half the Secchi depth. Multiple participants (scientists and crew of the research ship) took part. All Secchi depth and Forel Ule colour data collected were averaged at each station.

## RESULTS

**Fig. 1:**
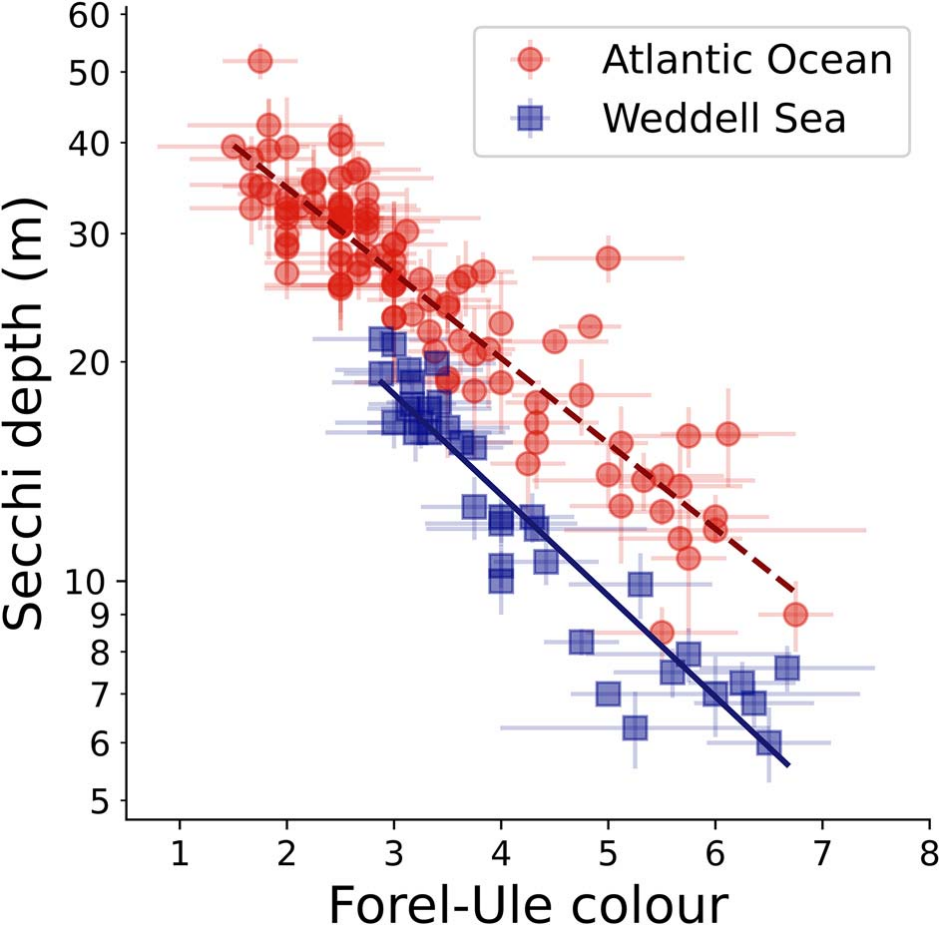
The relationship between Secchi depth (using a 30 cm white disk) and Forel-Ule colour (Novoa *et al.*, 2014) of water above the Secchi disk at half the Secchi depth in the Atlantic Ocean (circles, *N* = 102) and the Weddell Sea (squares, *N* = 36) using the same methods (Brewin *et al.*, 2023). Low values of Forel-Ule colour refer to bluer water, higher values greener water. Bars represent standard deviations for multiple samples at a station, from different participants. Regression lines (computed using IDL function ROBUST_LINEFIT.pro) are ${Z}_{\mathrm{SD}}={10}^{A\times{F}_U+B}$ (${Z}_{\mathrm{SD}}$ is the Secchi depth and ${F}_U$ the Forel-Ule colour) for the Atlantic Ocean (dashed line, $A=-0.117\ \left(\pm 0.005\right),B=1.774\ \left(\pm 0.011\right)$; Brewin *et al.*, 2023) and the Weddell Sea (solid line, $A=-0.138\ \left(\pm 0.009\right),\\ B = 1.672\ \left(\pm 0.020\right)$) and should not be extrapolated outside the range of data used in the fits.

True to the Southern-Ocean optical anomaly, we observed significantly bluer waters for similar levels of clarity at high latitudes when compared with low latitudes (Fig. 1). For the same colour, the Secchi depth was around 37% shallower in the Weddell Sea than in the Atlantic Ocean. These results confirm that optical anomalies can be detected and quantified with the human eye and simple optical tools. Deviations in the relationship between ocean colour and clarity for these two regions (Fig. 1) reflect differences in the composition, concentrations and spectral signatures of the optically active constituents (particles and substances) present in the water. Due to these anomalies, scientists should be cautious when interpreting ocean colour data from these two regions in the same way (e.g. by estimating phytoplankton concentration using satellite-derived band ratios of spectral remote-sensing reflectance and the same empirical algorithm).

## CONCLUSION

We have shown that a colour anomaly in the Weddell Sea can be identified visually. Our findings imply that spatial and temporal variations in aquatic ecosystems can be monitored optically using affordable methods. Furthermore, old records of Secchi depth and Forel-Ule colour collected over the last century (e.g. Wernand et al., 2013) may contain hidden clues on how ocean-colour anomalies have changed with climate.

## Data Availability

Data used in the publication are publicly available through the British Oceanographic Data Centre (https://doi.org/10.5285/f3198e10-faf3-1525-e053-6c86abc0d2f6 and https://doi.org/10.5285/142ca3c4-b400-2e79-e063-6c86abc09187).
